# APOBEC3G Interacts with ssDNA by Two Modes: AFM Studies

**DOI:** 10.1038/srep15648

**Published:** 2015-10-27

**Authors:** Luda S. Shlyakhtenko, Samrat Dutta, Jaspreet Banga, Ming Li, Reuben S. Harris, Yuri L. Lyubchenko

**Affiliations:** 1 Department of Pharmaceutical Sciences, College of Pharmacy, University of Nebraska Medical Center, Omaha, Nebraska, United States of America; 2 Department of Biochemistry, Molecular Biology, and Biophysics, Institute for Molecular Virology, Center for Genome Engineering, Masonic Cancer Center, University of Minnesota, 321 Church Street South East, 6-155 Jackson Hall, Minneapolis, Minnesota 55455 USA

## Abstract

APOBEC3G (A3G) protein has antiviral activity against HIV and other pathogenic retroviruses. A3G has two domains: a catalytic C-terminal domain (CTD) that deaminates cytidine, and a N-terminal domain (NTD) that binds to ssDNA. Although abundant information exists about the biological activities of A3G protein, the interplay between sequence specific deaminase activity and A3G binding to ssDNA remains controversial. We used the topographic imaging and force spectroscopy modalities of Atomic Force Spectroscopy (AFM) to characterize the interaction of A3G protein with deaminase specific and nonspecific ssDNA substrates. AFM imaging demonstrated that A3G has elevated affinity for deaminase specific ssDNA than for nonspecific ssDNA. AFM force spectroscopy revealed two distinct binding modes by which A3G interacts with ssDNA. One mode requires sequence specificity, as demonstrated by stronger and more stable complexes with deaminase specific ssDNA than with nonspecific ssDNA. Overall these observations enforce prior studies suggesting that both domains of A3G contribute to the sequence specific binding of ssDNA.

APOBEC3G (A3G) protein belongs to a family of DNA cytosine deaminases that can block HIV-1 replication in the absence of viral infectivity factor (Vif) protein^1^,^2^,^3^. Among these deaminases, A3G has the highest activity against HIV. A3G is a single polypeptide deaminase consisting of two domains: the catalytic C-terminal domain (CTD) and the non-catalytic N-terminal domain (NTD). The NTD does not have deaminase activity, but it is required for A3G to bind to both single-stranded DNA (ssDNA) and single-stranded RNA (ssRNA) and to package A3G into the HIV-1 capsid (paper^4^ and references therein). The NTD is positively charged, allowing A3G to interact with negatively charged nucleic acids. It is believed that the primary restrictive mechanism toward HIV-1 is deamination of viral cDNA; however, non-enzymatic mechanisms are also possible^5^,^6^. It has been proposed that A3G binding to viral RNA or single-stranded cDNA is a mechanism of non-enzymatic HIV-1 restriction^3^,^6^,^7^,^8^,^9^,^10^,^11^,^12^. This has been summarized as a road-block model in which A3G binds viral nucleic acid and blocks DNA synthesis^13^.

AFM imaging was used to directly demonstrate that A3G binds to ssDNA^14^,^15^,^16^. A3G can bind to ssDNA and ssRNA as a monomer^17^, but it self-assembles into dimers, trimers, and higher order oligomers in the nanomolar concentration range — an important property of A3G^15^,^17^. It is hypothesized that A3G oligomerization is a necessary step for the non-enzymatic restriction of HIV-1, including A3G encapsidation^3^,^6^,^7^,^8^,^9^,^10^,^11^,^12^. The aggregation propensity of A3G is associated with the NTD^15^,^17^,^18^,^19^, and the importance of the NTD to oligomerization is supported by the finding that the isolated CTD primarily exists in solution as a monomer^14^,^20^.

The structure of full length A3G is still unknown, though the 3D structure of CTD and NTD domains has been determined by NMR^20^,^21^ and X-ray crystallography^22^,^23^. Recently, a complex of CTD with sequence 5′-TTAACCCTTA-3′ containing tri-nucleotide 5′-CCC-3′ motif was crystallized, enabling the authors to identify residues in the protein involved in the CTD deamination reaction^24^. The authors also proposed a model for the full-size A3G in which the two domains are arranged in a side-by-side fashion, causing the entire A3G protein to be in an elongated ellipsoid shape.

Although abundant information on the biological activities of A3G is available, the interplay between the NTD and the CTD is still not fully understood. For example, the deamination of consecutive cytosine residues was reported to be influenced by ssDNA polarity^25^; however, according to our direct AFM imaging experiments, A3G binding to ssDNA was independent of polarity^19^. Additionally, the deamination activity of the isolated CTD is almost 100-fold less than the activity of full-size A3G, suggesting that the NTD contributes to enzymatic activity^17^,^20^. Another example is the observation that the deaminase reaction has strict sequence specificity, while A3G binding to ssDNA is not sequence specific. This was exemplified in the paper by Chelico *et al.*^26^, that showed that a 30-fold change in deaminase activity does not affect the binding efficiency of A3G to ssDNA.

Herein, we combined AFM topographic imaging with the ability of AFM to measure intermolecular interactions, termed AFM force spectroscopy, to characterize the interaction of full-length A3G with ssDNA containing different sequences. AFM imaging revealed that A3G is weakly dependent on DNA sequences when in complex with ssDNA. However, the use of AFM force spectroscopy revealed that A3G-ssDNA interactions are sequence-dependent, as evidenced by the strength and lifetimes of protein-ssDNA complexes. Additionally, these experiments directly showed that A3G binds to ssDNA substrates by two modes. Two-mode binding requires a long DNA substrate, as the use of ssDNA as short as 27 residues in length eliminated the ability of A3G to bind by two modes. We also discuss a model for the interaction between A3G and ssDNA.

## Results

### The role of oligonucleotide sequence on the formation of A3G-ssDNA complexes - AFM imaging

**Figure 1:**
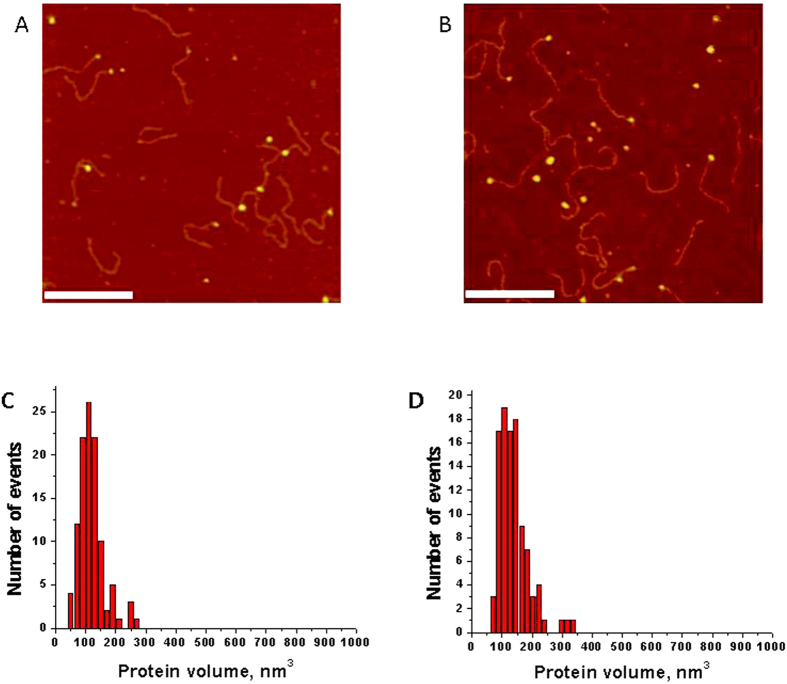
AFM topographic study of A3G and ssDNA complexes containing specific and nonspecific sequences. (**A**,**B**) are AFM images for specific and nonspecific substrates, respectively. The bar size = 200 nm. The complexes yields are 79.7 ± 2.2% for specific and 72.8 ± 5.6% for nonspecific sequences, respectively (t-test, p = 0.12). Errors are standard deviations. (**C**,**D**) are the protein volume histograms for complexes of A3G with specific and nonspecific sequences, respectively. The numbers of molecules counted are 107 for specific and 101 for non-specific complexes, respectively.

The AFM topographic imaging method was applied to characterize the binding of A3G to two ssDNA targets: one with CCC-motifs (deaminase specific) and a second without CCC-motifs (nonspecific) sequences (see Methods for full sequences). We used our DNA-hybrid methodology^14^,^15^,^19^ and designed two DNA substrates containing a 418 bp DNA duplex terminated with deaminase specific and nonspecific ssDNA sequences (Supplementary Figure S1). For both DNA substrates, the complexes were prepared at a 2:1 protein to DNA ratio. Typical AFM images for the complexes are shown in Fig. 1A for the deaminase specific complex and in Fig. 1B for the nonspecific sequence. These images demonstrate that both DNA substrates formed complexes with A3G, which are seen on the images as bright blobs at the ends of the ssDNA. We calculated the yield of the complexes to characterize the efficiency of complex formation. The analysis shown in Fig. 1 demonstrates that the yield of complexes with the specific sequence (79.7 ± 2.2%) is slightly higher than the yield for nonspecific sequences (72.8 ± 5.6%), and t-test gives p = 0.12 indicating to a non-significant difference between these values. We also characterized the A3G stoichiometry by measuring the protein volumes for both complexes. The histogram distributions show that complexes for specific sequence are primarily dimers, but monomers and higher order multimers also present as described^15^. The distribution for A3G bound to the nonspecific sequence is slightly wider than the distribution for the specific sequence, but still most representative species are the dimers. Therefore, we conclude that A3G binds to ssDNA in a weak sequence-dependent manner, which is consistent with the findings of Chelico *et al.*^25^,^24^ who demonstrated that a 30-fold decrease in A3G deaminase activity did not alter its binding efficiency.

### Probing A3G interactions with ssDNA

**Figure 2:**
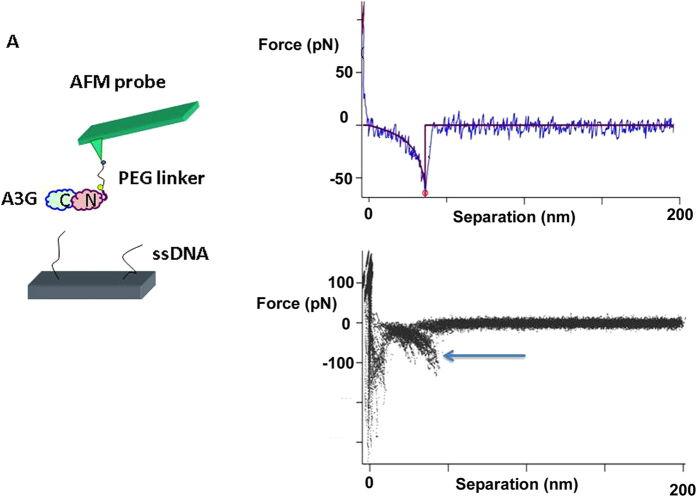
AFM force spectroscopy experiments for probing interactions of A3G with ssDNA. (**A**) Schematic of the probing experiment. A3G is covalently attached to the functionalized AFM tip via a PEG tether, and ssDNA is end-immobilized onto the mica surface that is functionalized with maleimide-silatranes via a thiol group. (**B**) A typical force-distance curve with the rupture event shown in blue. The red line represents the fit of the force curve using the WLC approach; contour length Lc = 47.8 nm, rupture force F_R _= 50 pN. (**C**) An overlay of more than one hundred rupture force events. The arrow points to the rupture event signature.

We used the AFM force spectroscopy approach to directly measure the interaction of A3G with specific and nonspecific ssDNA targets, as described^27^,^28^,^29^. In this approach, schematically shown in Fig. 2A, ssDNA covalently attached to the surface is probed by A3G immobilized onto the AFM tip via a flexible tether. The tether (PEG linker) provides orientational freedom to A3G during the interaction with the DNA target (see Materials and Methods section for specifics). One example of a typical individual force-separation curve is shown in Fig. 2B as a blue line. Initially, the protein and ssDNA are far away from each other (segment “a” on the force curve). Then, the AFM tip approaches the surface, and the DNA and protein interact during a 0.5 s dwell time to allow the complex to form (segment “b”). According to kinetic measurements of A3G-ssDNA complex formation, 50% of the complexes form in 1.1 ms^17^; therefore, a 0.5 s dwell time provides ample time for complex formation. The final step is the tip retraction step, during which the AFM tip is moved away from the surface, causing both the ssDNA and the PEG linker to stretch (segment “c”) and leading to A3G dissociation when the applied force is sufficient to rupture the complex (segment “d”).

More than 1000 events were collected for each pulling velocity, resulting in several hundred rupture events. The average yield of specific events was 5–10%. The specificity of interaction between A3G on the probe and ssDNA was verified by control experiments. In one control experiment, the functionalized mica surface without ssDNA attached was probed with A3G protein tethered to the AFM probe. The number of rupture events was less than 1%, with an estimated contour length of ~27 nm, as expected for the interaction of PEG-A3G protein with the functionalized surface. In a second control experiment, the functionalized AFM tip without A3G protein (PEG only) was used to probe the interaction with ssDNA tethered to the mica surface. The yield of the events was around 0.3%, which indicates that there are no interactions between ssDNA on the surface with the PEG linker attached to the probe.

We applied Eq. (1) to fit the force curves with the Worm-Like Chain (WLC) model. The fit in Fig. 2B validated the use of the WLC approach and allowed us to determine two major parameters: the contour length (Lc) and the rupture force (F) values. A superposition of more than hundreds of rupture events is shown in Fig. 2C. The overlay of force curves demonstrates the high reproducibility of the rupture events, which are indicated with an arrow. The Lc and F parameters for each force curve were obtained and analyzed.

**Figure 3:**
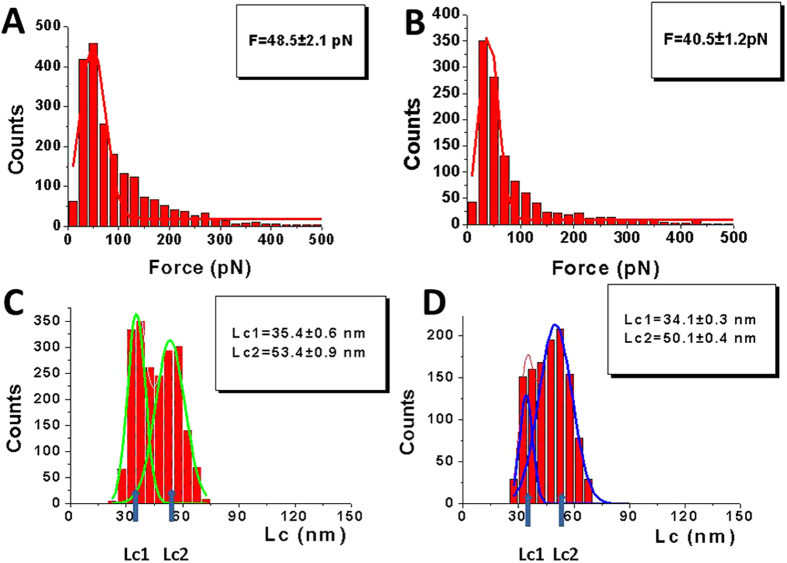
Quantitative analysis of the force spectroscopy data for probing events of specific and nonspecific sequences. (**A**,**B**) Rupture force histograms at all loading rates for specific and nonspecific sequences, respectively. The histograms are approximated with single Gaussians and the parameters of the fit are shown in the boxes. The T-test shows p = 0.11 indicating to a non-substantial differences in forces. (**C**,**D**) Contour length distribution histograms for specific and nonspecific sequences, respectively. Each distribution is approximated with two Gaussians. The fitting parameters are shown in the boxes with SEM indicated

Figure 3 shows the results for the analyses performed for the probing of specific and nonspecific ssDNA sequences. The distribution of the rupture forces for specific sequences is shown in Fig. 3A. There is a clear major peak on this distribution, and the approximation of the distribution with a Gaussian indicates a maximum value of 48.5 ± 2.1 pN. A similar analysis for the nonspecific sequence is shown in Fig. 3B. This distribution also has a definitive maximum, with a value of 40.5 ± 1.2 pN. A comparison shows that rupture forces for A3G interactions with specific ssDNA sequences are not strongly statistically higher than for nonspecific ssDNA sequences (p = 0.11).

We analyzed the contour length distributions for the specific and nonspecific ssDNA sequences. The histogram for the specific sequences (Fig. 3C) has two clearly identified maxima, which were approximated with two Gaussians at 35.4 ± 0.6 nm (Lc1) and 53.4 ± 0.9 nm (Lc2). A similar analysis for the nonspecific sequences is shown in Fig. 3D. The overall distribution is asymmetric, and the fit with two Gaussians indicates maxima at 34.1 ± 0.3 nm and 50.1 ± 0.4 nm. Note that the maxima values for Lc1 and Lc2 are very similar for the specific and nonspecific sequences. However, the populations of events for Lc1 and Lc2 are different for the two sequences. For the specific interaction, the number of events at Lc1 and Lc2 are similar; however, for the nonspecific interaction, the number of events at Lc2 is 4 fold higher than the number of events at Lc1.

In order to obtain more detailed information about the specific and nonspecific interactions of A3G with ssDNA, we analyzed the rupture forces for the contour lengths Lc1 and Lc2 separately. For this analysis, we chose subsets of contour lengths to avoid overlapping values in the histograms in Fig. 3C,D. The range between 30–40 nm was selected for the short contour lengths (Lc1), and the range between 50–60 nm was selected for the long contour lengths (Lc2).

**Figure 4:**
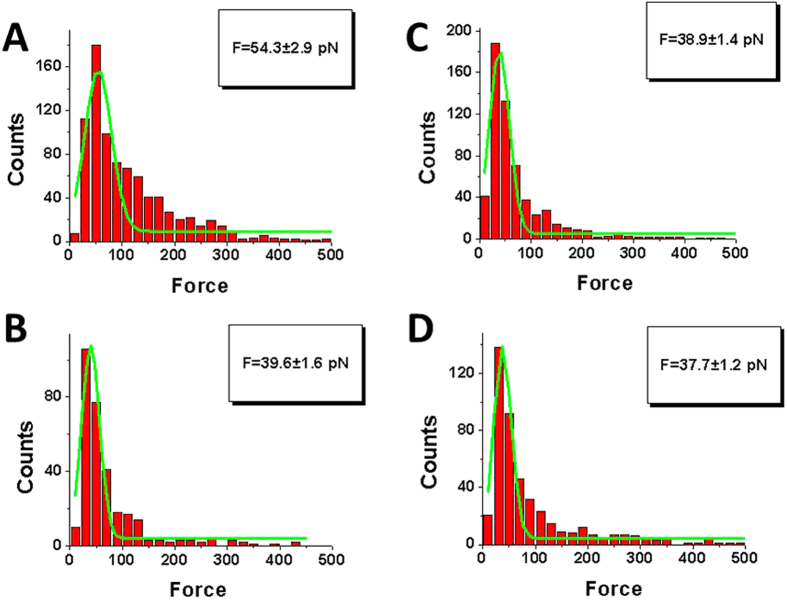
The force distribution histograms at all loading rates for subsets defined by the contour lengths Lc1 and Lc2. The first peak on histograms are approximates with single Gaussian. The fitting parameters are shown in boxes with SEM indicated. Histograms (**A**,**C**) correspond to distributions of rupture force for contour length Lc1 for specific and nonspecific sequences, respectively. T-test shows p«0.001 meaning substantial differences. Histograms (**B**,**D**) correspond to rupture force distributions for contour length Lc2 for specific and nonspecific sequences, respectively. The histograms are approximated with single Gaussians. The parameters of the fit with SEM are shown in the boxes.

The analysis of the selected subset data for Lc1 during the interaction of A3G with specific and nonspecific sequences is shown in Fig. 4A,C, respectively. The distributions for rupture forces were fitted with Gaussians and the maxima force values for specific and nonspecific DNA sequences were 54.3 ± 2.9 pN and 38.9 ± 1.4 pN, respectively. The values are very different; indeed, according to the t-test p«0.001. These results indicate that the interaction of A3G with ssDNA is associated with rather strong sequence specificity, as exemplified by the short rupture events.

A similar analysis was performed for the selected subsets for Lc2 for both DNA sequences. The results for rupture force distributions are shown in Fig. 4B,D. The Gaussians for the specific and nonspecific sequences have maxima at 39.6 ± 1.6 pN and 37.7 ± 1.2 pN, respectively. These values are very close, suggesting that the DNA sequence does not affect interactions at long rupture events.

### Dynamic force spectroscopy analysis of ssDNA-A3G complexes

**Figure 5:**
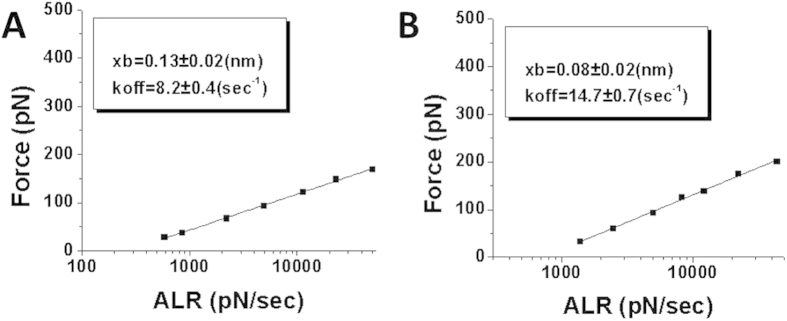
DFS plots for interactions of A3G with specific (**A**) and nonspecific (**B**) ssDNA sequences. The plots were obtained by interpolating entire datasets with eq. (3) as described in^31^. k_off_ along with x_b_ values with the fitting errors calculated from the linear plots are shown in the boxes.

To analyze the dynamics of the A3G-ssDNA interaction, we used the dynamic force spectroscopy (DFS) approach in the framework of the Bell-Evans model^30^ in which probing experiments are performed at different pulling rates. The data were analyzed as described in^31^ wherein the linear DFS plot was generated over the entire dataset. Supplementary Figure S4 shows such datasets for the probing experiments for A3G protein with specific (A) and nonspecific (B) ssDNA substrates. The linear plots for both experiments are shown in Fig. 5A,B. The points in these plots correspond to intersections of the graphs with the most populated areas in the data sets indicated in Fig. S4 by crosses. DFS plots enabled us to determine the off-rate constant (k_off_) that characterizes the stability of the A3G-ssDNA complexes. The k_off_ values were obtained using Eq. 2 (Methods section): specific = 8.2 ± 0.4 s^−1^, and nonspecific = 14.7 ± 0.7 s^−1^. These data indicate that the interaction of A3G with specific sequences leads to the formation of more stable complexes compared to those formed by nonspecific sequences. Along with off-rates, the DFS plot provides distances to transition state x_b_. These values for specific and non-specific substrates are 0.13 ± 0.02 nm and 0.08 ± 0.02 nm, respectively. These parameters are different suggesting that the protein structure in the complexes depends on ssDNA sequence.

**Figure 6:**
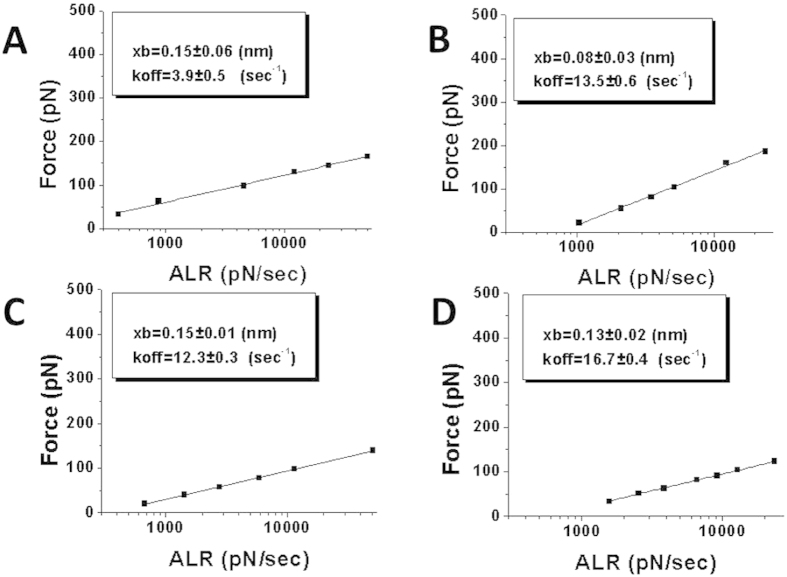
DFS analysis for the contour length subsets Lc1 and Lc2 for the interactions of A3G with ssDNA. (**A**,**D**) show the DFS plots for the Lc1 contour length for specific and nonspecific sequences, respectively. (**C**,**B**) show the DFS plots for the Lc2 contour length for specific and nonspecific sequences, respectively. The k_off_ along with x_b_ values with the fitting errors calculated from the linear plots are shown in the boxes.

Next, we performed a similar DFS analysis separately for contour length subsets corresponding to Lc1 and Lc2, as described in the section above. The DFS plots for Lc1 A3G interactions with specific and nonspecific sequences are shown in Fig. 6A,D, respectively. Their corresponding k_off_ values are 3.9 ± 0.5 s^−1^ and 16.7 ± 0.4 s^−1^, respectively. These results demonstrate that the short contour length, Lc1, defines sequence specific interactions. The k_off_ values for complexes corresponding to Lc2 are very similar (Fig. 6C,B), 12.3 ± 0.3 s^−1^ and 13.5 ± 0.6 s^−1^ for specific and nonspecific sequences, respectively.

The rupture force values for the interaction of A3G with specific and nonspecific sequences for all of the events along with k_off_ and values for the distance to transition state x_b_ are summarized in Table 1.

## Discussion

### The effect of DNA sequence on A3G-ssDNA complex formation

The results described above reveal that topographic imaging does not show clear sequence specificity of A3G interacting with ssDNA. Indeed, the difference in the complex yield (Fig. 1) is rather small, thereby explaining why the effect was not detected with the use of traditional EMSA techniques in^25^. However, the effect of the sequence is getting pronounced when AFM force spectroscopy was used to probe the A3G-ssDNA interactions. There is a tendency of forming slightly stronger and more stable complexes with specific vs. non-specific sequences (Table 1). The difference is getting apparent for the subsets corresponding to the short contour length Lc1 (see Table 1 data for Lc1), with significant differences observed in the stability defined by the off-rate values (~4 fold) and strength (~1.4 fold) of specific interactions compared to nonspecific interactions.

### Different interacting modes of A3G with ssDNA

One unexpected finding of the force probing experiments is the appearance of two types of rupture events, resulting in the contour lengths Lc1 and Lc2, as shown in Fig. 3C,D. One can argue that such a bimodal distribution can be due to an asymmetric attachment of A3G protein through N-terminus (see Material and Methods section). To test this hypothesis, we designed an A3G-Cys mutant in which a cysteine residue was placed at the C-terminus for the protein immobilization on the AFM tip. Other Cys residues were replaced with alanines. For the protein immobilization we used another bifunctional PEG tether with the same PEG length (see the Material and Methods section for details) that contained a maleimide group that specifically reacts with Cys resides. The results for the deaminase specific 69 nt ssDNA probed with A3G-cys are shown in Supplementary Figure S3. The contour length distribution is similar to the distribution shown in Fig. 3C, with two well-defined peaks. Additionally, the positions of the peaks are very well correlated: Lc1 = 35.4 ± 0.6 nm, Lc2 = 53.4 ± 0.9 nm for NHS-primary amine-N-terminus attachment, and Lc1 = 37.5 ± 1.4 nm and Lc2 = 53.8 ± 2.5 nm for maleimide-cysteine-N-terminus attachment. These data show that the bimodal distribution does not depend on the attachment position being N or C terminal. Indeed the flexible PEG linker allows the protein to change orientation freely before interaction with the ssDNA target.

A bimodal distribution could be linked to the two-domain structure of A3G, and to test this hypothesis we performed AFM probing experiments with the one-domain A3A protein and the same DNA substrates. These results are shown in supplemental Figure S5, in which an overlay of more than hundreds of events (Fig. S5A) is shown, along with the contour length distribution (Fig. S5B). The distribution is mono-modal, which supports our hypothesis about the nature of the bimodal distribution of A3G protein.

**Figure 7:**
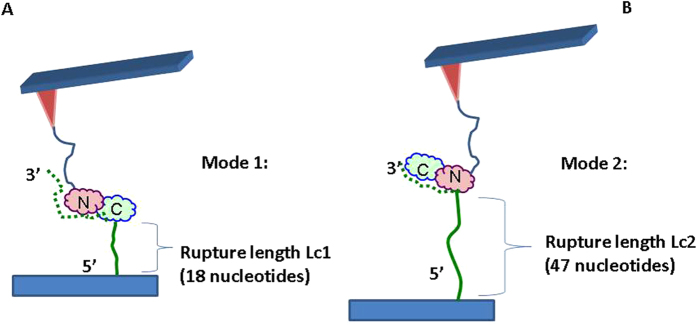
Schematics for the two possible binding modes of A3G protein. (A) Mode 1 requires an extra ~30 nt with A3G located closer to the 5′-ssDNA end, resulting in rupture events at short contour lengths. (**B**) Mode 2 occurs when A3G is located close to the 3′-ssDNA end, resulting in rupture events at longer contour lengths. The dashed line indicates schematically ssDNA segments involved in the complex formation with A3G to distinguish them with non-complexed segments shown in solid lines.

Based on these results we propose the model depicted in Fig. 7 to explain the AFM probing data for A3G. In this model, the two arrangements of A3G are shown schematically as a symbol with two lobes (C) and (N), representing the CTD and NTD domains respectively. A3G protein is attached to the AFM tip at the N-terminus via a flexible PEG tether. ssDNA is tethered to the functionalized mica surface at its 5′terminus. Therefore, in mode 1 (Fig. 7A), the distance between the protein and the 5′-end is short, corresponding to the contour length Lc1. In mode 2 (Fig. 7B), the protein orientation is different, and the distance between A3G and the 5′-DNA end is longer, corresponding to the contour length Lc2. The dashed lines in the figure indicate the ssDNA segments in complexes with A3G to distinguish them from non-interacting segments of ssDNA shown in solid line. According to Fig. 3C,D, the difference between Lc2 and Lc1 is ~18 nm, which corresponds to ~30 nt in the DNA length. This suggests that in mode 2, the interacting ssDNA segment is almost 2-fold shorter. Our two-mode A3G-DNA binding model is consistent with a model proposed by Chelico *et al.*^17^, in which A3G has two different orientations while interacting with ssDNA, termed active and non-active orientations. Moreover, the authors identified a 30 nt dead zone, which is needed for the active A3G orientation. The 30 nt difference between Lc2 and Lc1 is intriguingly close to the dead zone size, suggesting that mode 1 may correspond to the active A3G orientation.

To clarify the role of DNA length on A3G-ssDNA interactions, we performed experiments by probing shorter DNA. Supplemental Figure S7 shows the contour length histogram for probing experiments of A3G with a 27 nt ssDNA. This is a narrow, single-peak distribution, indicating that A3G loads on short DNA with one mode. These data suggest that much longer DNA is required for the two-mode A3G loading process.

### Two-mode A3G loading on ssDNA and sequence specificity

Our results in Fig. 3 demonstrate that two-mode binding of A3G to ssDNA occurs for both specific and nonspecific sequences. Therefore, this property of A3G does not required the preferred target sequence 5′-CCC. However, the type of interaction mode is influenced by sequence specificity. First, DNA sequences have a greater effect in mode 1 binding, as observed by the 4-fold difference in the complex lifetime of specific sequences compared to the lifetime of nonspecific sequences (Table 1). Second, in mode 1 binding, the rupture forces are higher for specific sequences than for nonspecific sequences. Comparatively, mode 2 binding is rather insensitive to the DNA sequence. Finally, the sequence also influences the partition between the two loading modes, as observed in Fig. 3. Both types of complexes are formed with equal probabilities for specific sequences (Fig. 3C); however, mode 2 is preferable for nonspecific sequences (Fig. 3D).

Experiments performed with a short DNA substrate (27 nt vs. 69 nt; Supplementary Figure S6) demonstrate that only one A3G interaction mode occurs. This finding suggests that two-mode binding requires a long DNA substrate. Studies of A3G deamination of ssDNA with different lengths^25^ showed that ssDNA oligonucleotides as short as 9 nt can be deaminated. Therefore, A3G in both binding modes is capable of deamination. However, according to Chelico *et al.*^25^, the deamination reaction efficiency decreases with oligonucleotide length. The maximal A3G deamination activity corresponds to the ssDNA length of ~70 nt, for which we observed a two-mode A3G interaction. Therefore, we speculate that mode 1 binding provides the most efficient A3G deamination; however, additional data are needed to support this assumption. Mode 1 binding also suggests that the majority of the long ssDNA target is complexed with A3G. The accommodation of long ssDNA by A3G is consistent with the single FRET studies of A3G-DNA interactions that also showed that ssDNA binding to A3G is accompanied by compaction of the ssDNA geometry^32^. The FRET results are also consistent with the two-mode model proposed by the authors earlier in^17^. Altogether, our results demonstrate that the interaction of A3G with ssDNA is an intricate process in which both the NTD and CTD contribute to complex formation.

## Materials and Methods

### DNA-substrate preparation

The procedure to assemble the hybrid DNA substrate for the AFM topographic studies of A3G-DNA complexes is described in the details^15^,^19^ and schematics in Supplementary Figure S1. The hybrid DNA substrate, comprised of a 418 bp DNA duplex, was ligated to a single-stranded oligonucleotide that produced a 69 nucleotide (nt) overhang.

The following 69 nt overhangs were made:

deaminase specific sequence:

**5**′**TACGTGTAGGAATTATATTAAAGAGAAAGTGAAACCCAAAGAATGAAAACCCAAATGTTAGAATTGTTA3**′

non-deaminase specific sequence: **5**′**TACGTGTAGGAATTATATTAAAGAGAAAGTGAAAAGAAGAGAATGAAAAAGAAGATGTTAGAATTGTTA3**′

27 nt deaminase specific sequence was also used:

**5**′**TTTTAAAGAGAAAAACCCAAAAGTGAA3**′.

These oligonucleotides, terminated with thiols at their 5′ ends (Integrated DNA Technologies, Inc. Coralville, Iowa), were used for AFM force spectroscopy experiments. The 5′ thiol-modified oligos were prepared by treating them with DTT followed by ethyl acetate extraction to remove DTT, as recommended by the manufacturer (Integrated DNA Technologies, Inc. Coralville, Iowa).

### Protein purification

Detailed procedures for the protein purification and deamination activity assays for A3G and A3A have been reported^15^,^33^.

For force spectroscopy experiments imidazole has been removed from the storage buffer of the protein by dialyzing the protein using Slide-A-Lyzer dialysis cassettes (Thermo Fisher) into 50mM Tris-HCl, pH8.0, 300mM NaCl, 0.1% Triton X-100 and 10% Glycerol buffer.

#### A3G-Cys mutant preparation

A3G surface cysteines C139, C243, C261, C308, C321 and C356 were identified by A3G CTD structure (3IR2) and A3G NTD modeling. These cysteines were replaced with alanines by site-directed mutagenesis in pcDNA3 1-A3G-mycHis plasmid^15^,^33^. A new cysteine codon TGC was introduced into the 3′-end of 6xHis codons by overlap-extension PCR. The encoded recombinant A3G 6A-mycHisCys (A3G-Cys) was purified as described above. The deaminase activity of the A3G and A3G-Cys are presented in Supplementary Figure S2.

### AFM imaging of A3G-DNA complexes

APS-mica was used for the AFM sample preparation, as described in^15^. For the APS-DNA complex formation procedure, A3G was mixed in a 2:1 ratio with the hybrid DNA substrate in a buffer containing 50 mM HEPES, pH 7.5, 100 mM NaCl, 5 mM MgCl_2_, and 1 mM DTT, and incubated for 15 min at 37 °C. Next, 5 μl of the complex was deposited onto the surface for 2 min, followed by rinsing with deionized water (AquaMax, CA). Images were acquired in the tapping mode in air on the Multimode Nanoscope IV system (Bruker, Santa Barbara, CA). TESPA silicon based AFM probes (Bruker) with the spring constant ~40 nN/nm and a resonance frequency of ~300 Hz were used.

### Data analysis

The DNA length, protein volume (cross section option), and the number of complexes were calculated using the FemtoScan program (Advanced Technologies Center, Moscow, Russia), as described in^15^,^19^. The data for the DNA lengths and protein volumes were assembled into histograms using the Origin 8.5 program (Origin Lab, Northampton, MA). The 1.3 conversion coefficient was used to convert the measured protein volume in nm^3^ into protein mass in kDa, as described in^19^.

### AFM force spectroscopy experiments

#### A3G immobilization to the AFM probes

Two different protocols were used for the covalent attachment of A3G to the AFM probe via a flexible bifunctional tether.

##### The covalent attachment through a primary amine at the N-terminus (NHS-chemistry)

The protocol is based on the functionalization of the AFM tips with a bifunctional PEG tether containing a succinamide group at the ends (SVA-PEG-SVA), as described in^28^,^29^. Briefly, the AFM probe (MSNL, Bruker, CA) was cleaned with methanol followed by UV irradiation for 1 hour. The AFM probe was then immersed into a 167 μM APS solution for 30 min to functionalize the surface. The probe was rinsed with water, followed by DMSO. The amino-functionalized AFM probe was then incubated with a bifunctional SVA-PEG-SVA linker in a DMSO solution for 3 hours. 2 mM SVA-PEG-SVA (MW = 3400 g/mol, Laysan Bio Inc. Arab, Al) was used to prevent possible bridging of the PEG linker. After 3 hours of incubation, the probe was washed with PBS buffer, pH 7.8, to remove DMSO, then immersed into a 5 nM A3G solution and incubated overnight to covalently bind the NHS group of the PEG linker on the probe to the primary amine at the N-terminus of A3G protein. A 1 M Tris buffer was used to remove the excess non-covalently bound protein and to block unreacted amino groups on the surface of the probe. Finally, the probe was immersed into a probing buffer containing 50 mM HEPES, 100 mM NaCl, 5 mM MgCl_2,_ and 1 mM DTT.

##### The covalent attachment through cysteine at the C-terminus of A3G-cys (maleimide chemistry)

A3G-cys was prepared by treating it with DTT to disrupt possible disulfide bonds, followed by ethyl acetate extraction to remove DTT, as recommended by the company (Integrated DNA Technoloqies, Inc. Coralville, Iowa).

The AFM probe was functionalized with APS, as described above, and incubated with a 500 μM bifunctional SVA-PEG-MAL linker (MW = 3400 g/mol, Laysan Bio Inc. Arab, Al) in a DMSO solution for 3 hours. The probe was washed with PBS buffer, pH 7.1, to remove DMSO and immersed into a 5 nM A3G-cys solution and incubated overnight to covalently bind the maleimide group from the PEG linker on the probe to the cysteine residue at the C-terminus of A3G-cys. A 1 M Tris buffer was used to remove excess non-covalently bound protein and to block unreacted amino groups on the surface of the probe. Finally, the probe was immersed into probing buffer.

#### Modification of the mica surface

APS-mica was used as the initial substrate for surface modification, as described in^28^,^29^,^34^. Briefly, a freshly cleaved mica surface was amino functionalized with a 167 μM APS water solution for 30 min. Next, APS mica was treated with 500 μM sulfo-GMBS (N-γ-maleimidobutyryl-oxysulfosucciminide ester) from Thermo Fisher Scientific Inc. (Waltham, MA) in DMSO for 3 hours to bring the maleimide groups to the surface. The surface was then washed with PBS buffer, pH 7.1, and incubated with the 30 μM 5′-thiol-modified 69 nt or 27 nt oligos overnight. The samples were washed with 1 M Tris buffer to remove unbound oligos and to block unreacted amino groups on the surface. The surface was then immersed into the probing buffer.

#### Single-molecule force spectroscopy experiments

The experiments were performed on the MFP3D instrument (Asylum Research, Santa Barbara, CA). The force distance curves for interactions between ssDNA and A3G protein were collected at room temperature in the probing buffer containing 50 mM HEPES, 100 mM NaCl, 5 mM MgCl_2,_ and 1 mM DTT.

The spring constant of the functionalized AFM probe (MSNL from Bruker) was measured using the thermal noise method and was in the range of 20–40 pN/nm. The parameters for the force spectroscopy experiments were set as follows: trigger force at contact = 100 pN, dwell time = 0.5 s, and approach rate = 500 nm/s. For dynamic force spectroscopy experiments, the pulling velocity was varied in the range of 100 nm/s–3000 nm/s. More than 1000 events were collected for each pulling velocity, resulting in several hundred rupture events.

The A3G protein was predominantly a monomer at the chosen concentration, as was shown in^16^.

### AFM force spectroscopy data analysis

The force-distance (F-D) curves were analyzed with the data processing software Igor Pro 6.31, provided by Asylum Research (Santa Barbara, CA). The force curves were fitted with the Worm-Like Chain (WLC) model^35^, using the following equation:


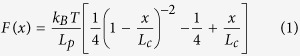


where F(x) is the force at the distance of x, k_B_ is the Boltzmann constant, T is the absolute temperature, and L_p_ and L_c_ are the persistence length and the contour length, respectively. The WLC model was chosen because it properly describes the elasticity of a polypeptide chain^36^,^37^. The persistence length was varied to produce the best fitting curve and evaluated as a variable parameter along with the contour length. Each experiment consisted of hundreds of specific force distance curves were analyzed and fitted to produce a set of rupture forces (F), contour lengths (L_c_), and persistence lengths (L_p_). The L_c_ and F values were used to construct histograms for contour length and rupture forces that were fitted with the Gaussian function using the Origin 8.5 software (Origin Lab., Northampton, MA).

Apparent loading rates (ALR) at different retraction speeds were calculated using the following equation^28^:


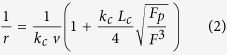


where F_p _= k_B_T/L_p_, k_c_ is the spring constant (N/m), v is the tip velocity (pulling rate; m/s), F is the rupture force, and r is the apparent loading rate (pN/s).

For each force curve, the apparent loading rate value “r” (ALR) was obtained and the dependence of the rupture force on the log (ALR) for the entire dataset was generated (Fig. S4). The Dynamic force spectroscopy (DFS) analysis was performed in the framework of the Bell-Evans model^30^, using the following equation for the dependence of the rupture force (F) on the log (ALR):


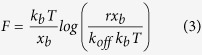


The DFS linear plots were obtained as described in^31^ in which the entire dataset without dividing it into subsets was used for the interpolation using eq. (3). From the linear plots, the off-rate constants, k_off_, and the distances to the transition state, x_b_, were determined.

## Additional Information

**How to cite this article**: Shlyakhtenko, L. S. *et al.* APOBEC3G Interacts with ssDNA by Two Modes: AFM Studies. *Sci. Rep.* **5**, 15648; doi: 10.1038/srep15648 (2015).

## Supplementary Material

Supplementary Information

## Figures and Tables

**Table 1:** The results of the force spectroscopy analyses for A3G interactions with specific and non-specific ssDNA targets.

Specific DNA sequence	Force max (pN)^a^	k_off_ (s^−1^)^b^	x_b_ (nm)^c^	Lc max (nm)^d^
Overall rupture events	48.5 ± 2.1	8.2 ± 0.4	0.13 ± 0.02	
Subset for Lc1	54.3 ± 2.9	3.9 ± 0.5	0.15 ± 0.06	Lc1–35.4 ± 0.6
Subset for Lc2	39.6 ± 1.6	12.3 ± 0.3	0.15 ± 0.01	Lc2–53.4 ± 0.9
**Non-specific DNA sequence**				
Overall rupture events	40.5 ± 1.2	14.7 ± 0.7	0.08 ± 0.02	
Subset for Lc1	38.9 ± 1.4	16.7 ± 0.4	0.13 ± 0.02	Lc1–34.1 ± 0.3
Subset for Lc2	37.7 ± 1.2	13.5 ± 0.6	0.08 ± 0.03	Lc2–50.1 ± 0.4

^a^Maximum rupture force values corresponding to the Gaussian fit of the force distributions.

^b^Off-rate constant values obtained from the DFS plots.

^c^The distance to transition state x_b_ obtained from the DFS plots.

^d^Maximum contour length values corresponding the Gaussian fit of the contour lengths distributions.
