# Supplementary material for: APOBEC3G Interacts with ssDNA by Two Modes: AFM Studies
Source: Sci Rep. 2015 Oct 27;5:15648. doi: 10.1038/srep15648 (PMC4621513; doi:10.1038/srep15648)
Supplement: Supplementary Information [file srep15648-s1.pptx]

## Slide 1
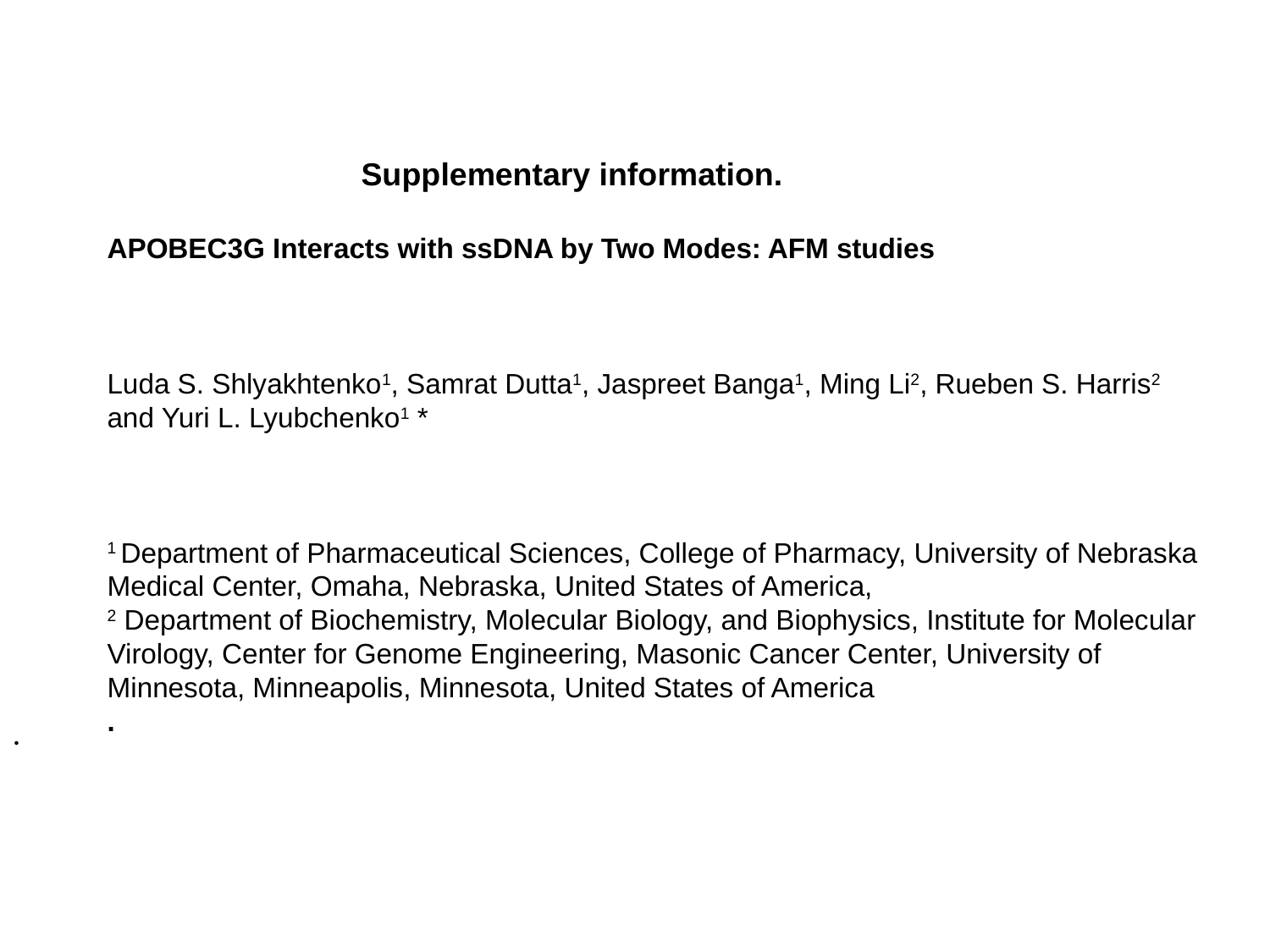

Supplementary information.
APOBEC3G Interacts with ssDNA by Two Modes: AFM studies
Luda S. Shlyakhtenko1, Samrat Dutta1, Jaspreet Banga1, Ming Li2, Rueben S. Harris2 and Yuri L. Lyubchenko1 *
1 Department of Pharmaceutical Sciences, College of Pharmacy, University of Nebraska Medical Center, Omaha, Nebraska, United States of America,
2 Department of Biochemistry, Molecular Biology, and Biophysics, Institute for Molecular Virology, Center for Genome Engineering, Masonic Cancer Center, University of Minnesota, Minneapolis, Minnesota, United States of America
.
.

## Slide 2
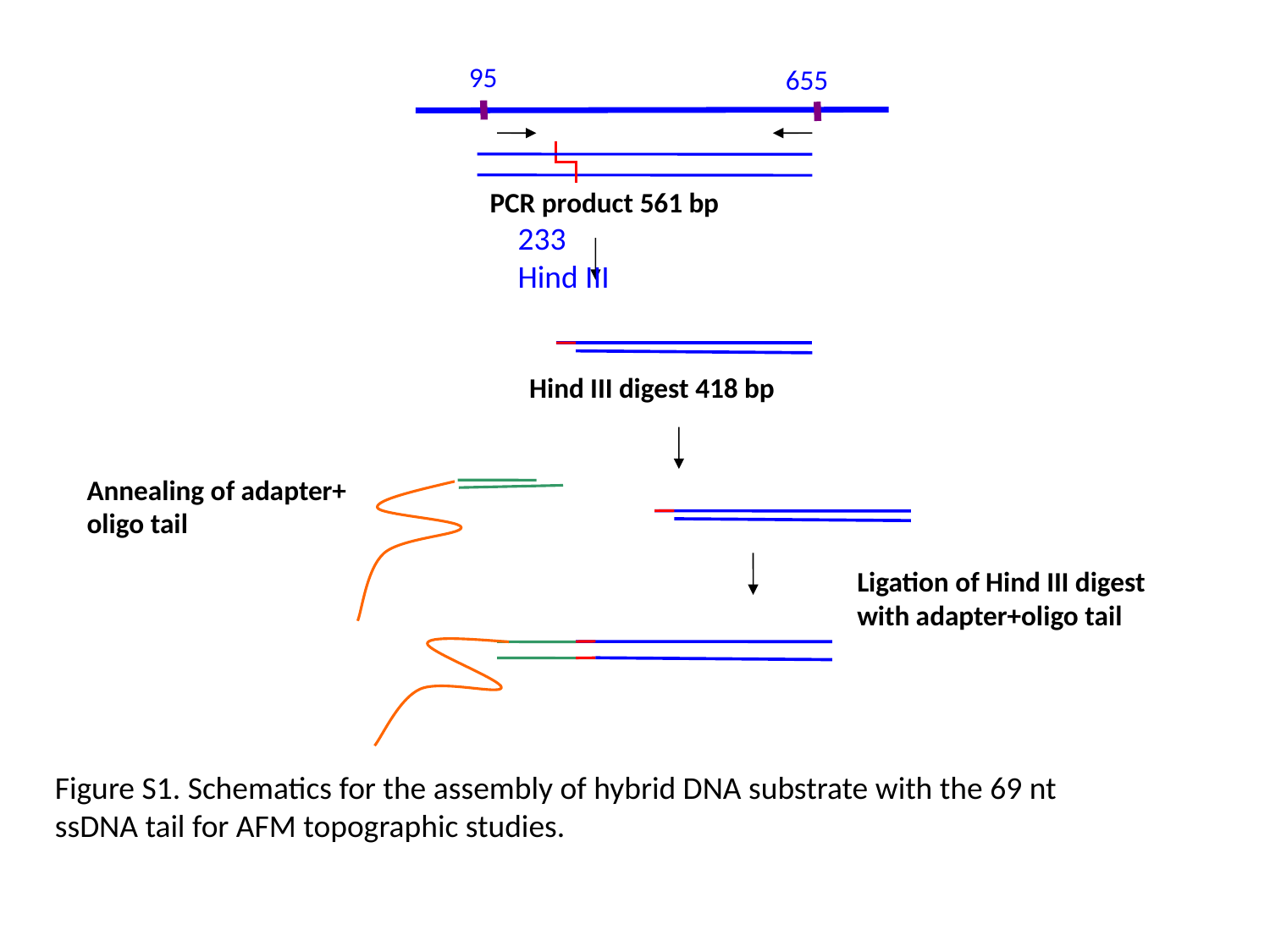

95
655
PCR product 561 bp
233
Hind III
Hind III digest 418 bp
Annealing of adapter+ oligo tail
Ligation of Hind III digest with adapter+oligo tail
Figure S1. Schematics for the assembly of hybrid DNA substrate with the 69 nt ssDNA tail for AFM topographic studies.

## Slide 3
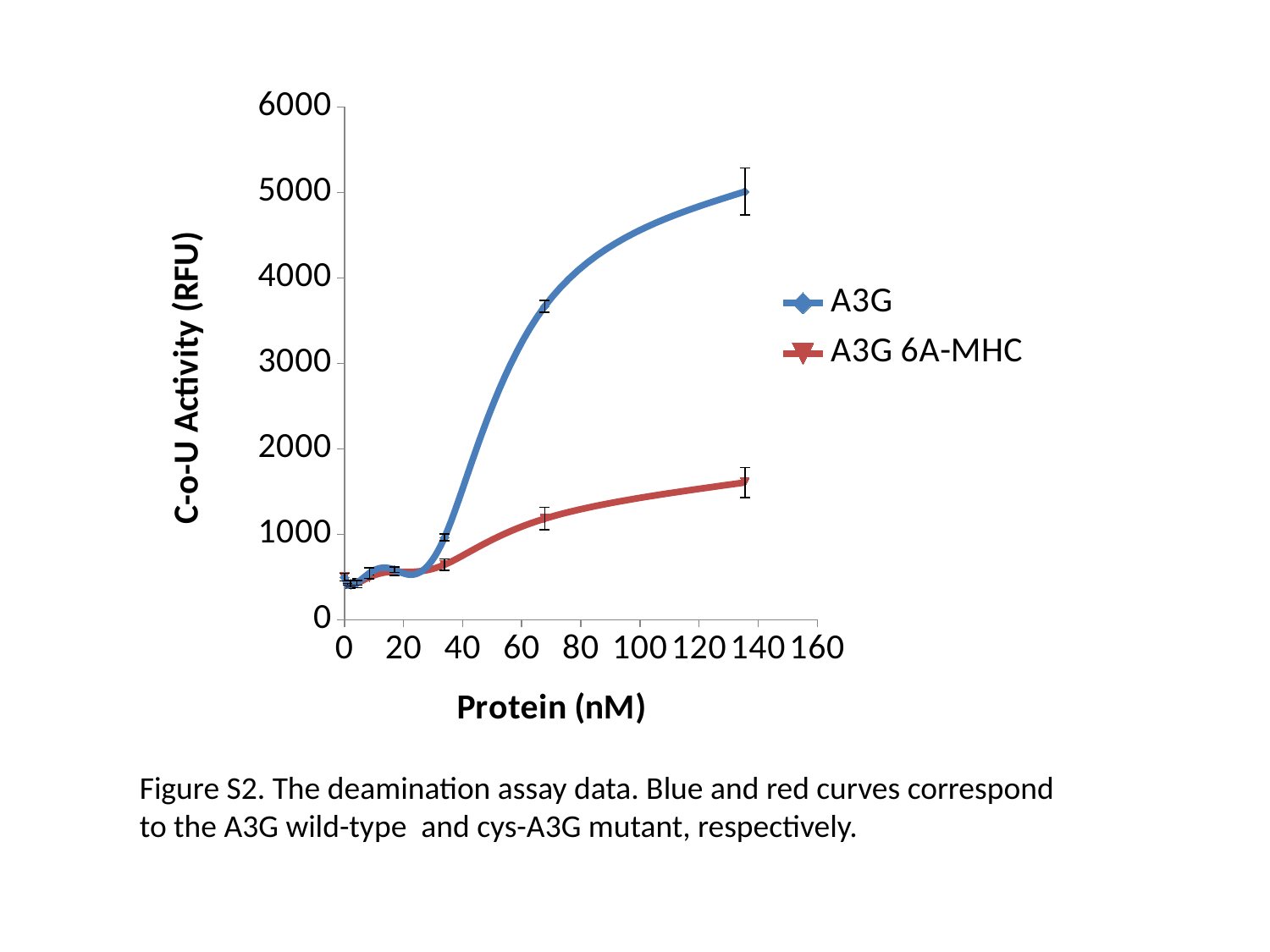

### Chart
| Category | A3G | A3G 6A-MHC |
|---|---|---|Figure S2. The deamination assay data. Blue and red curves correspond to the A3G wild-type and cys-A3G mutant, respectively.

## Slide 4
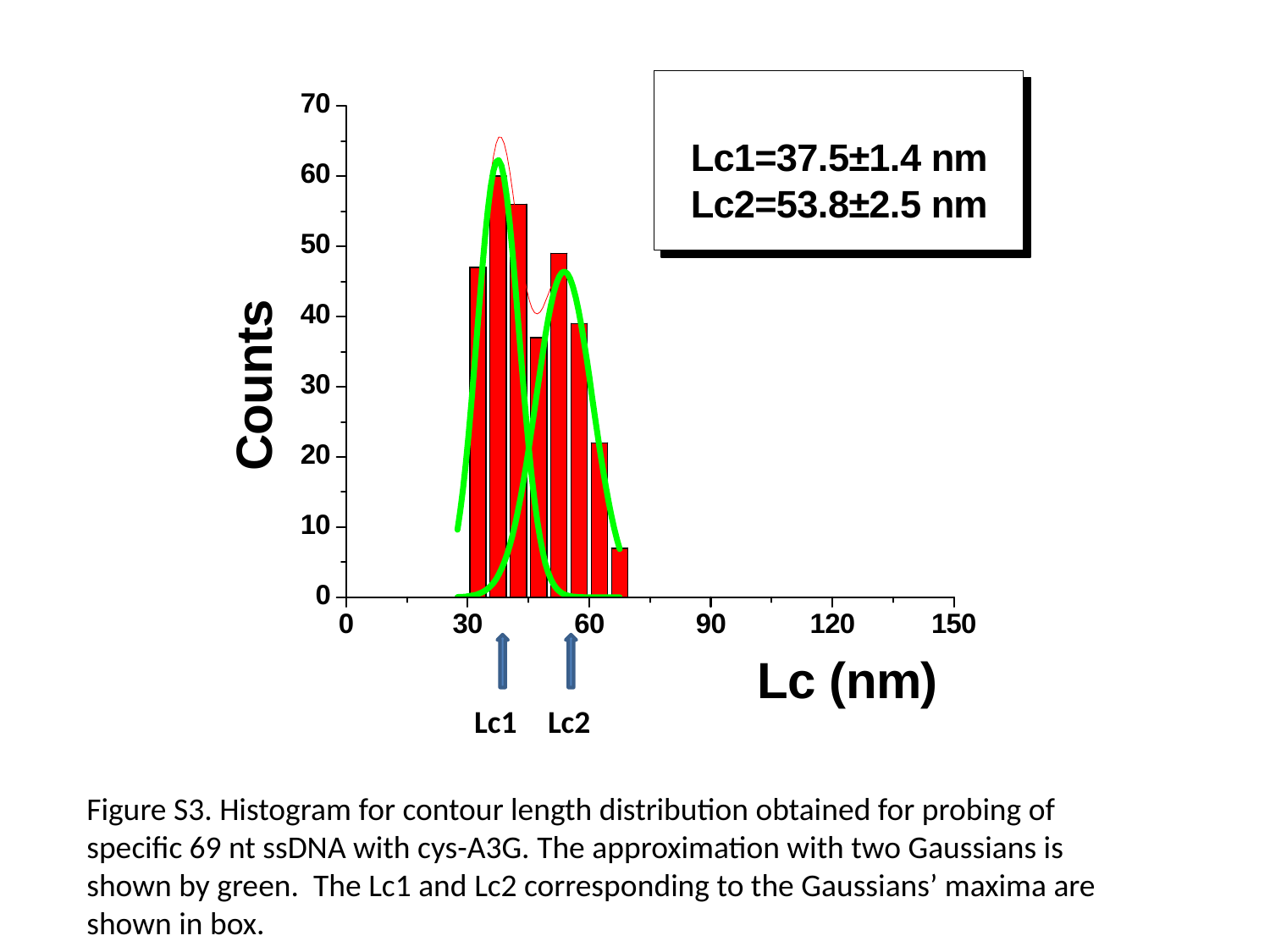

Lc1
Lc2
Figure S3. Histogram for contour length distribution obtained for probing of specific 69 nt ssDNA with cys-A3G. The approximation with two Gaussians is shown by green. The Lc1 and Lc2 corresponding to the Gaussians’ maxima are shown in box.

## Slide 5
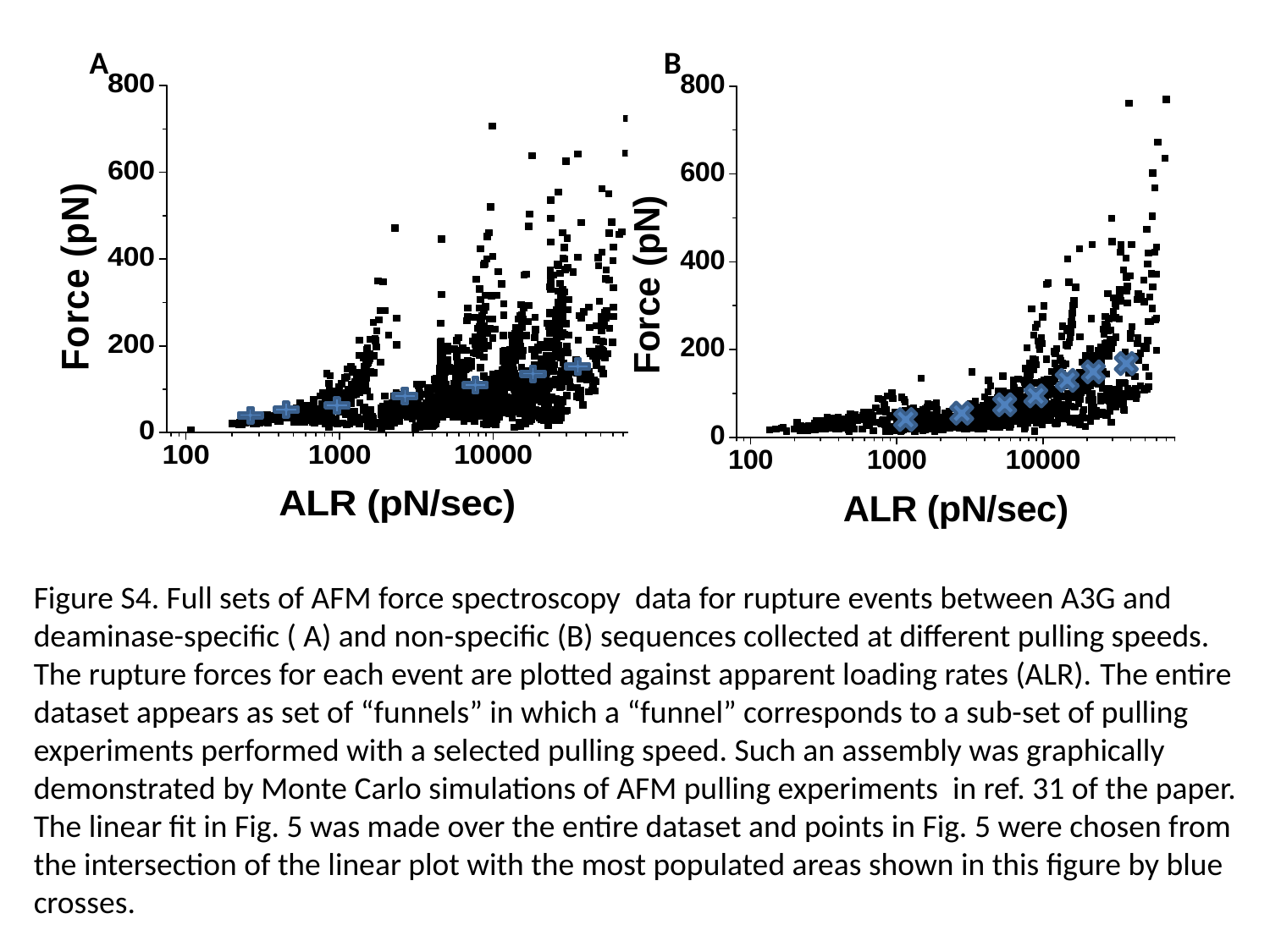

A
B
Figure S4. Full sets of AFM force spectroscopy data for rupture events between A3G and deaminase-specific ( A) and non-specific (B) sequences collected at different pulling speeds. The rupture forces for each event are plotted against apparent loading rates (ALR). The entire dataset appears as set of “funnels” in which a “funnel” corresponds to a sub-set of pulling experiments performed with a selected pulling speed. Such an assembly was graphically demonstrated by Monte Carlo simulations of AFM pulling experiments in ref. 31 of the paper. The linear fit in Fig. 5 was made over the entire dataset and points in Fig. 5 were chosen from the intersection of the linear plot with the most populated areas shown in this figure by blue crosses.

## Slide 6
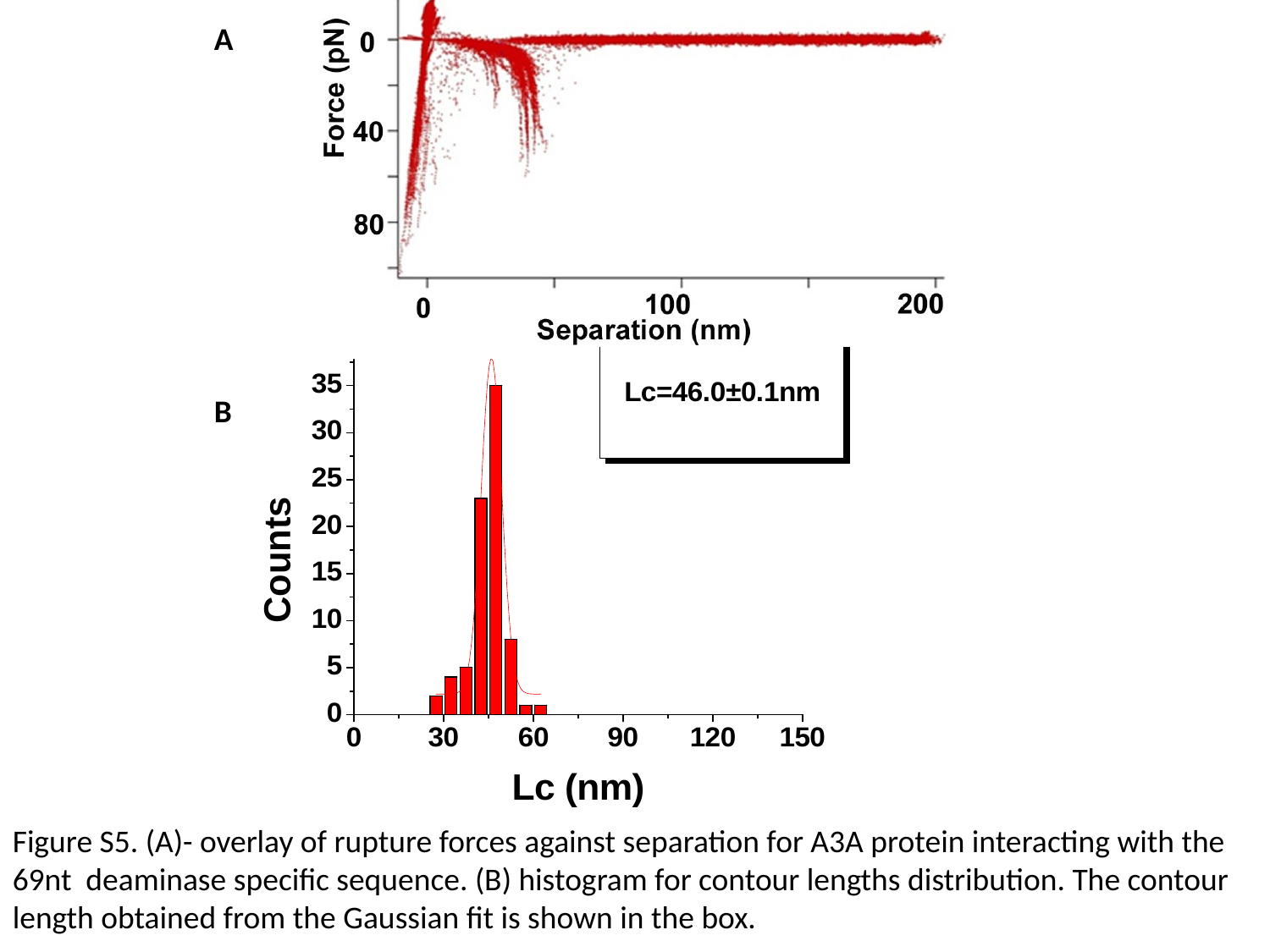

A
B
Figure S5. (A)- overlay of rupture forces against separation for A3A protein interacting with the 69nt deaminase specific sequence. (B) histogram for contour lengths distribution. The contour length obtained from the Gaussian fit is shown in the box.

## Slide 7
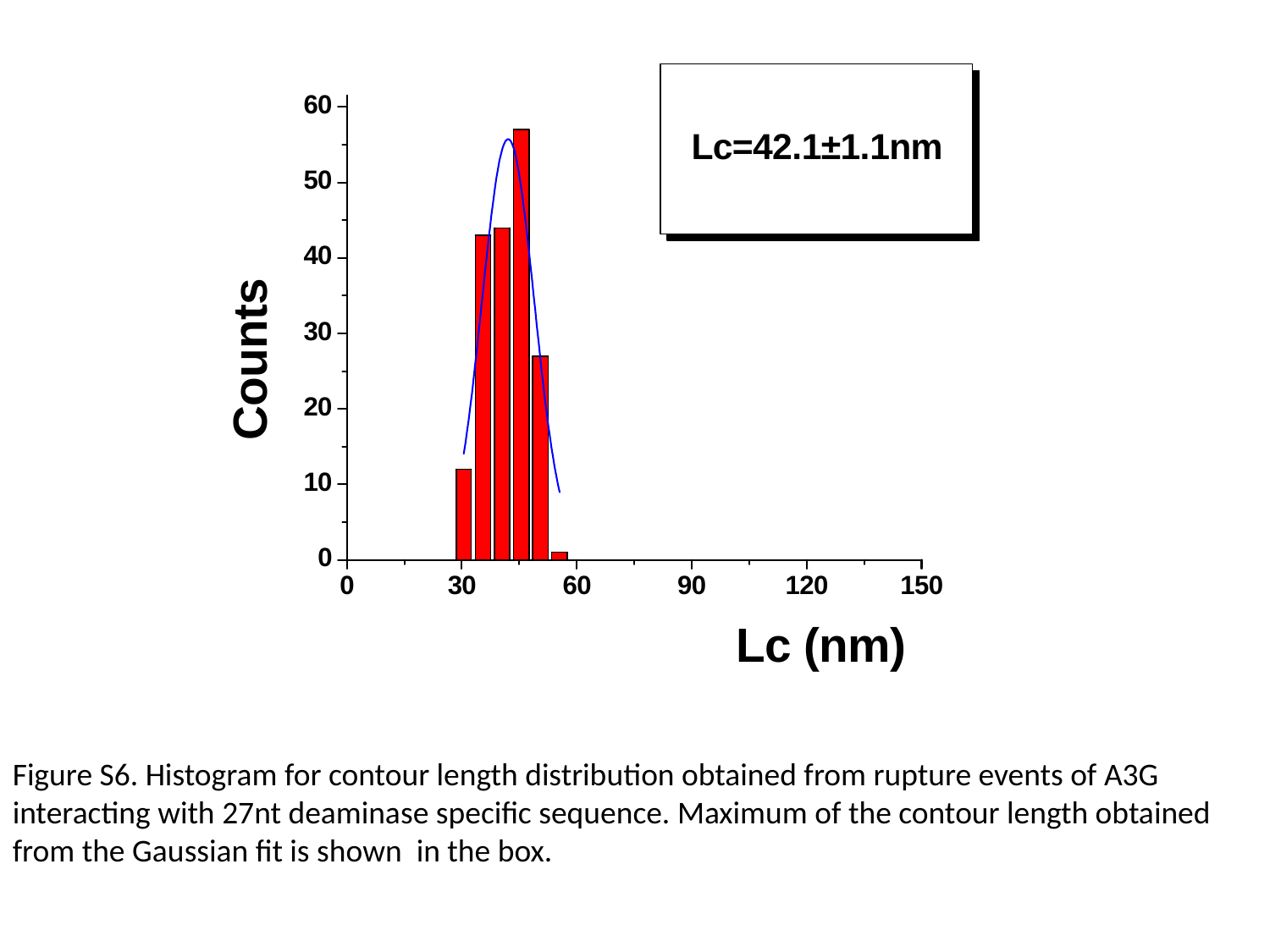

Figure S6. Histogram for contour length distribution obtained from rupture events of A3G interacting with 27nt deaminase specific sequence. Maximum of the contour length obtained from the Gaussian fit is shown in the box.

## Slide 8
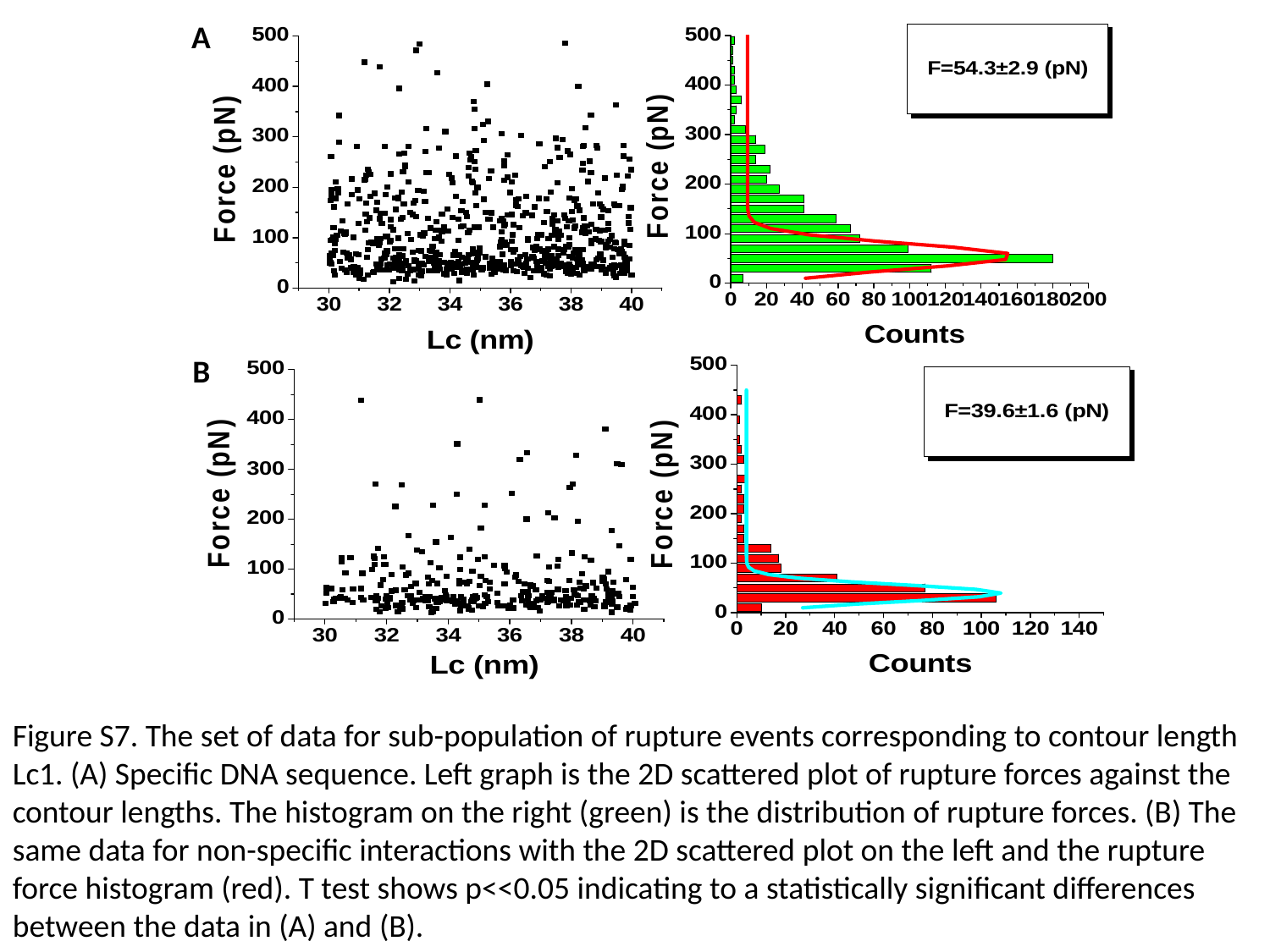

A
B
Figure S7. The set of data for sub-population of rupture events corresponding to contour length Lc1. (A) Specific DNA sequence. Left graph is the 2D scattered plot of rupture forces against the contour lengths. The histogram on the right (green) is the distribution of rupture forces. (B) The same data for non-specific interactions with the 2D scattered plot on the left and the rupture force histogram (red). T test shows p<<0.05 indicating to a statistically significant differences between the data in (A) and (B).

## Slide 9
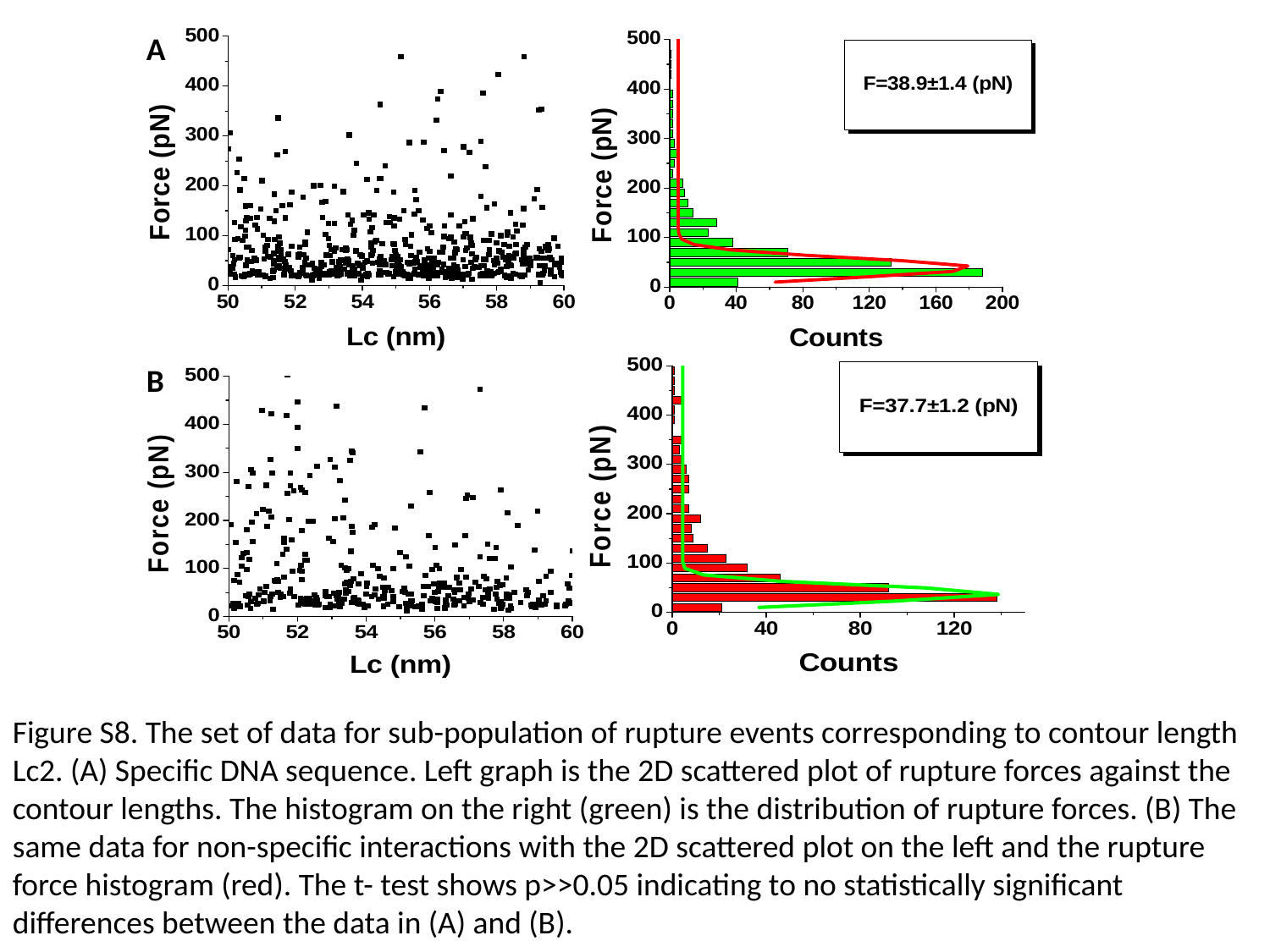

A
B
Figure S8. The set of data for sub-population of rupture events corresponding to contour length Lc2. (A) Specific DNA sequence. Left graph is the 2D scattered plot of rupture forces against the contour lengths. The histogram on the right (green) is the distribution of rupture forces. (B) The same data for non-specific interactions with the 2D scattered plot on the left and the rupture force histogram (red). The t- test shows p>>0.05 indicating to no statistically significant differences between the data in (A) and (B).
